# Supplementary material for: Phytosphingosine induces systemic acquired resistance through activation of sphingosine kinase
Source: Plant Direct. 2021 Sep 30;5(10):e351. doi: 10.1002/pld3.351 (PMC8483070; doi:10.1002/pld3.351)
Supplement: Supplementary file 1 — Table S1. Primers used in this study for real‐time qRT‐PCR. [file PLD3-5-e351-s001.docx]

Supplemental Data

Table S1**.** Primers used in this study for real-time qRT-PCR.

| Gene | Strand | Sequences |
| --- | --- | --- |
| *β-Actin* | Forward | TCACAGAAGCTCCTCCTAATCC |
|  | Reverse | GGGAAAGAACAGCCTGAATG |
| *NtMC2* | Forward | tccagctgaaactggtgaag |
|  | Reverse | tacaattgtgatccggcaac |
| *NtRbohD* | Forward | CGTCGTACAGTGGTCCGTTA |
|  | Reverse | CGTACGCATCATCATTGGAC |
| *NtRbohF* | Forward | CAGCTTGATCGGACTCGTTC |
|  | Reverse | CCGTCTTTAGCGAGCTTTGAG |
| *NtACS1* | Forward | GGAGAAGAAGCAGCAATGGA |
|  | Reverse | CTCGAACGAGTGGTGAATGAG |
| *NtACS2* | Forward | CCTTCAAATCCACTAGGCACTC |
|  | Reverse | GGGCTGATCAAAGACCGTAG |
| *NtACS4* | Forward | ACTAAGCCGAAACGAGCTTG |
|  | Reverse | TTCCATGACACTGACGAAGC |
| *CAT1* | Forward | CGCCATGCTGAGAAGTATCC |
|  | Reverse | AAAGCGTTCTTGCCTGTCTG |
| *CAT2* | Forward | CTACGATTCGATTGCTGCTG |
|  | Reverse | GGCCAGGTCTTGGTTACATC |
| *MnSODmi* | Forward | GGAGGTCACATTAACCACTCG |
|  | Reverse | CAGCACCTTCTGCATTCATC |
| *CuZnSODc* | Forward | CATGGTGCTCCTGAAGATGAG |
|  | Reverse | GATTGTGGACCAGCAAGAGG |
| *APXc* | Forward | TGTTCCCTTTCACCCTGGTAGAG |
|  | Reverse | CGTTCCTTGTGGCACCTTCC |
| *GSTF* | Forward | CGAAGCGCAATTGTCTAAGG |
|  | Reverse | ACACCATGCACTCACACGAG |
| *PR-1a* | Forward | TTGAGATGTGGGTCGATGAG |
|  | Reverse | CCTAGCACATCCAACACGAA |
| *PR-3* | Forward | AGGAACGACGGTAGATGTCC |
|  | Reverse | TCCTACGGGCAGTATCATCA |
| *PR-4b* | Forward | ATGGCTGGACTGCTTTCTGT |
|  | Reverse | CTCACTGTTGCTTGAGTTCCTG |
| *PR-6* | Forward | TAGTTTCCTTGCTCTCGTCCTC |
|  | Reverse | ACGTGGACAAACCCCATAAG |
| *PR 5 (TLP)* | Forward | GTCGTAATCTCAGATGCACAGC |
|  | Reverse | AGTAGGCCCACATGATCCAG |
| *PR 5 (OSM)* | Forward | GGTGACTGTGGTGGAGTCCTA |
|  | Reverse | GGCGAAAGTCATCGGTATGT |
| *SAR 8.2* | Forward | GATGTCTAAGGCGGCTGTTC |
|  | Reverse | TTCGTATCCAACCCGGTATC |
| *NPRI* | Forward | GCTGTAGCATATTGCGATGC |
|  | Reverse | GCAACATGCAGCACTGTGTA |
| *Ppn* | Forward | TGAACGCATATTGCACTTCC |
|  | Reverse | CATCTCCTCCACCGACTACAC |
| *SphK* | Forward | CTGTCGGTGAATCTTGTACTGC |
|  | Reverse | ATATCAGCTACAAGACCCCAGG |
| *LCB1* | Forward | ttgattgtgaggccaggata |
|  | Reverse | acaccttcatccgcaacaat |
| *LCB2* | Forward | ccgatcagaactgcagaaga |
|  | Reverse | caatcactacggccacattc |
| *ACER3* | Forward | cttgtgaatgccctcagaca |
|  | Reverse | aagcatttcccagaccatgg |
| *NCDase* | Forward | gtSaacatgatgggRtatgSt |
|  | Reverse | tgtgaRgccatRcMagcatc |
| *DPL* | Forward | AAGGAGATCATGGAGGCATC |
|  | Reverse | ACGCAGAACATCATTGACTTC |
| *NtORM1* | Forward | CTTCTCATGGCTTCTTGTTC |
|  | Reverse | TTACGAGTGAGCTGTTTCCC |
| *NtORM2* | Forward | GTGGATGGCTGTGCTCTCTG |
|  | Reverse | TGGGAGTAAGCTGCTTTCCA |
