# Supplementary material for: Phytosphingosine induces systemic acquired resistance through activation of sphingosine kinase
Source: Plant Direct. 2021 Sep 30;5(10):e351. doi: 10.1002/pld3.351 (PMC8483070; doi:10.1002/pld3.351)
Supplement: Supplementary file 2 — Figure S1. Effect of PHS on ROS accumulation in guard cells of tobacco leaves. A, ROS accumulation in guard cells was determined using confocal laser scanning microscopy (CLSM) after staining with 50 μM DCFH‐DA. The CLSM images of DCF fluorescence (green) were merged with the bright‐field images in the third column. Scale bars = 20 μm. B, Quantitation of DCF fluorescence intensity was calculated using ImageJ software. Figure S2. Effect of PHS on ROS accumulation in guard cells of Ppn‐inoculated tobacco leaves. The CLSM images of DCF fluorescence (green; DCFDA staining) and chlorophyll (Chl) autofluorescence (red) were determined at the indicated time after PHS treatment under Ppn inoculation. Both CLSM images were merged in the third column while the images in the bright field were merged in the fourth column. White boxes denote nuclei. Images are representative of three independent experiments with more than ten CLSM images at each indicated time. Scale Bars = 20 μm. Figure S3. Effect of PHS on expression profiles of ACS isoforms in Ppn‐inoculated tobacco leaves. Transcription levels of NtACS gene family members NtACS1 (left panel), NtACS2 (middle panel), and NtACS4 (right panel) at the indicated times after treatment with 1 μM PHS in Ppn‐infected tobacco leaves. Transcription levels are expressed relative to the reference gene β‐actin after qRT‐PCR. Ethylene levels and relative mRNA expression levels are expressed as means ± SD. An asterisk indicates a significant difference in PHS‐treated cases or PHS and Ppn inoculation co‐treated cases from mock‐treated cases (one asterisk (P < .05) or two asterisks at the same time point (P < .01)). [file PLD3-5-e351-s002.pptx]

## Slide 1
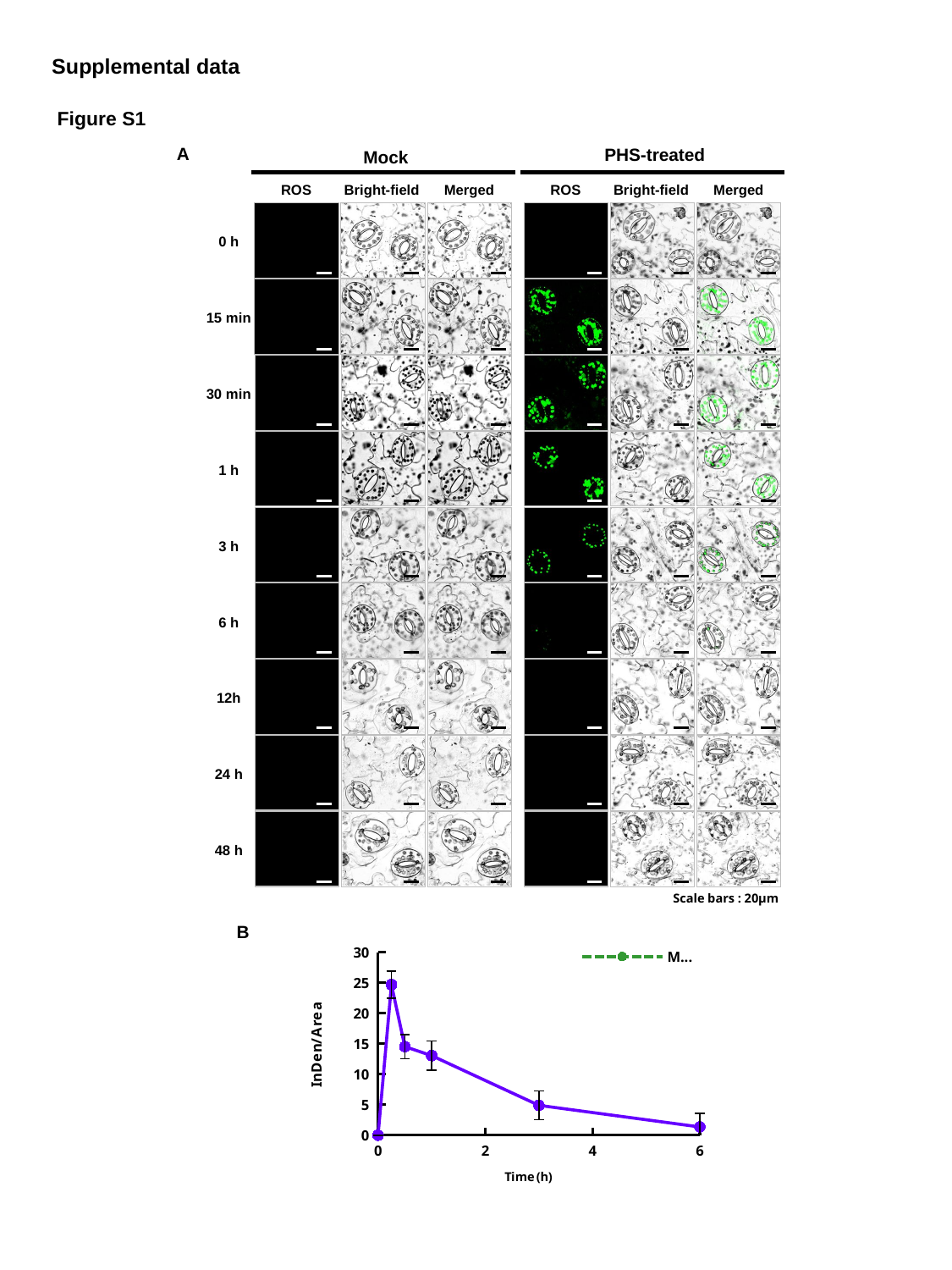

Supplemental data
Figure S1
A
 PHS-treated
 Mock
ROS
Bright-field
Merged
ROS
Bright-field
Merged
0 h
15 min
30 min
1 h
3 h
6 h
12h
24 h
48 h
Scale bars : 20µm
B
### Chart
| Category | Mock | PHS |
|---|---|---|

## Slide 2
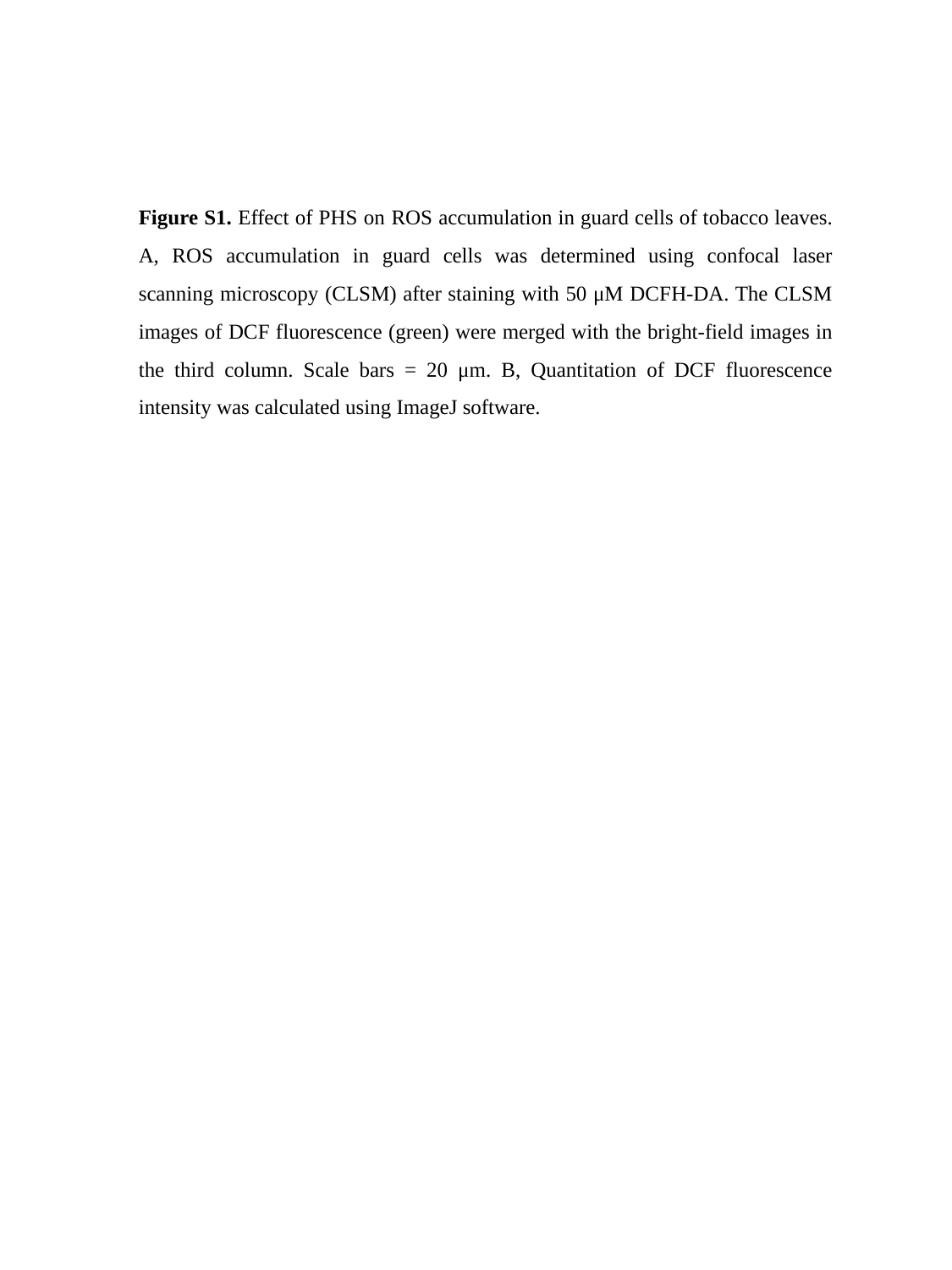

Figure S1. Effect of PHS on ROS accumulation in guard cells of tobacco leaves. A, ROS accumulation in guard cells was determined using confocal laser scanning microscopy (CLSM) after staining with 50 μM DCFH-DA. The CLSM images of DCF fluorescence (green) were merged with the bright-field images in the third column. Scale bars = 20 μm. B, Quantitation of DCF fluorescence intensity was calculated using ImageJ software.

## Slide 3
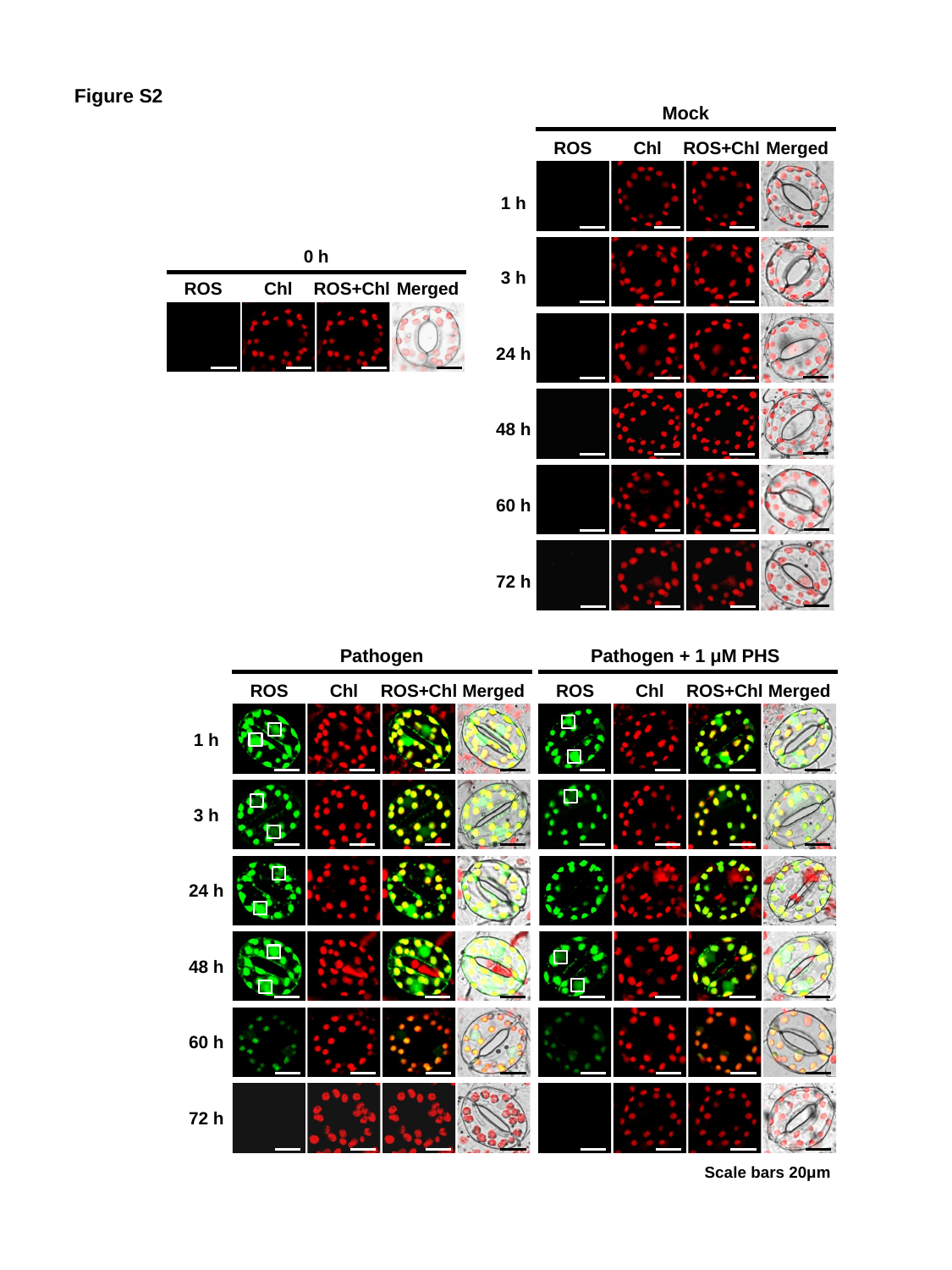

Figure S2
Mock
ROS
Chl
ROS+Chl
Merged
1 h
3 h
24 h
48 h
60 h
72 h
0 h
ROS
Chl
ROS+Chl
Merged
Pathogen
Pathogen + 1 μM PHS
ROS
Chl
ROS+Chl
Merged
ROS
Chl
ROS+Chl
Merged
1 h
3 h
24 h
48 h
60 h
72 h
Scale bars 20μm

## Slide 4
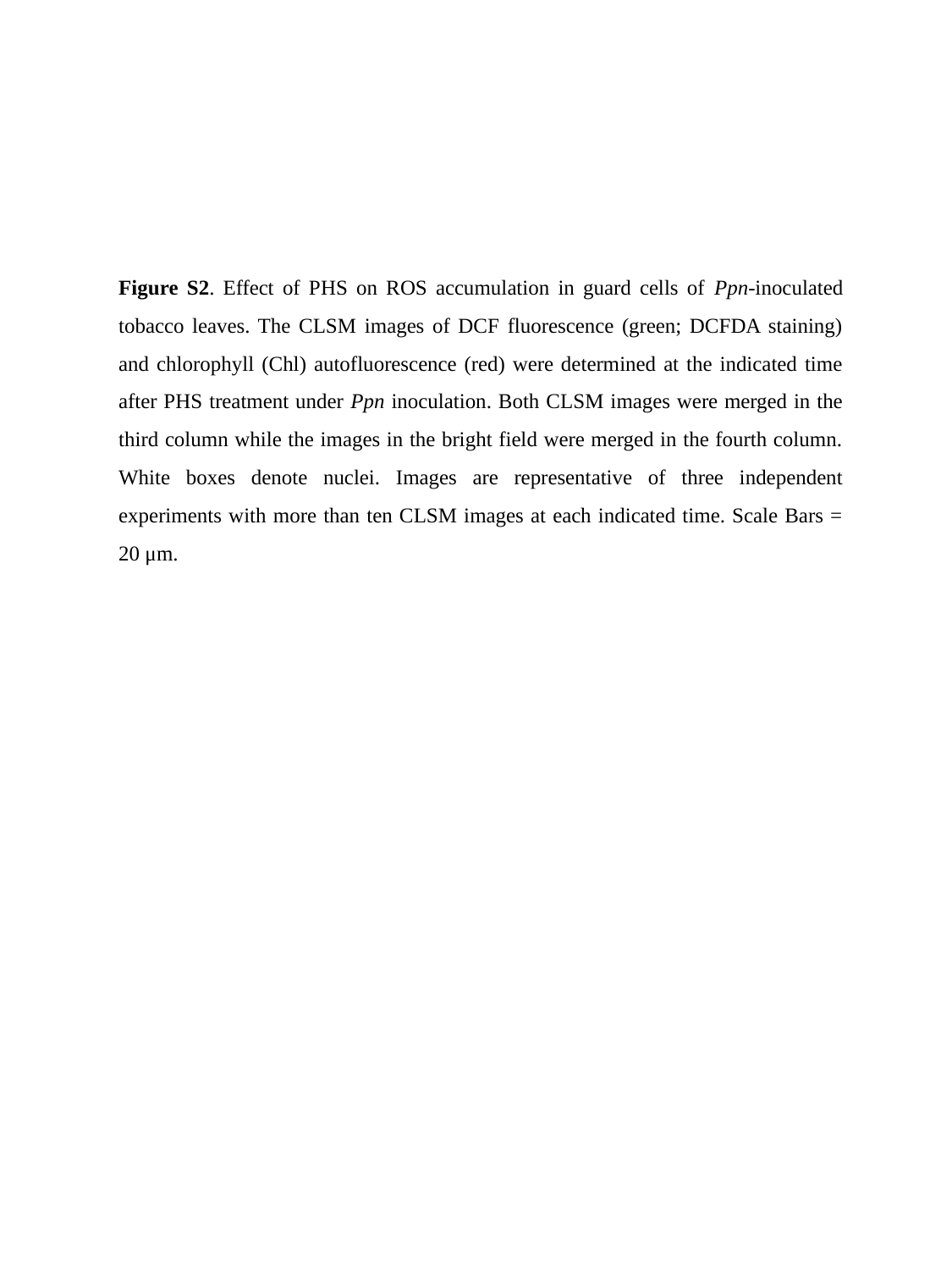

Figure S2. Effect of PHS on ROS accumulation in guard cells of Ppn-inoculated tobacco leaves. The CLSM images of DCF fluorescence (green; DCFDA staining) and chlorophyll (Chl) autofluorescence (red) were determined at the indicated time after PHS treatment under Ppn inoculation. Both CLSM images were merged in the third column while the images in the bright field were merged in the fourth column. White boxes denote nuclei. Images are representative of three independent experiments with more than ten CLSM images at each indicated time. Scale Bars = 20 μm.

## Slide 5
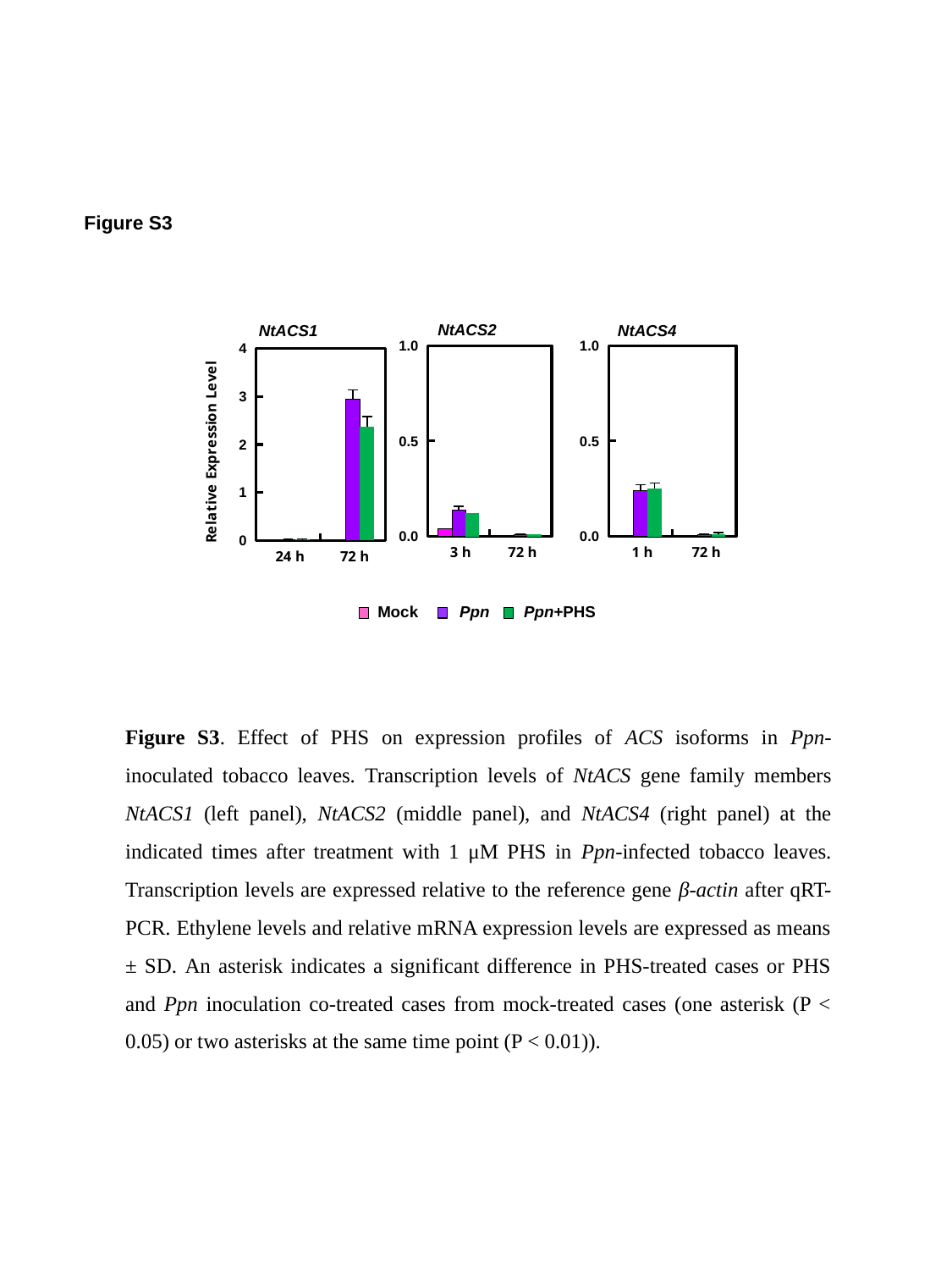

Figure S3
NtACS2
NtACS4
NtACS1
### Chart
| Category | Mock | Ppn | Ppn+PHS |
|---|---|---|---|
| 24 h | 0.005803904117298043 | 0.02 | 0.02 |
| 72 h | 0.005621983779289218 | 2.94 | 2.37 |
### Chart
| Category | Mock | Ppn | Ppn+PHS |
|---|---|---|---|
| 3 h | 0.04 | 0.14 | 0.12 |
| 72 h | 0.00336874359236223 | 0.01 | 0.01 |
### Chart
| Category | Mock | Ppn | Ppn+PHS |
|---|---|---|---|
| 1 h | 0.00204235824408875 | 0.24 | 0.25 |
| 72 h | 0.00193970064950389 | 0.01 | 0.01 | Mock Ppn Ppn+PHS
Figure S3. Effect of PHS on expression profiles of ACS isoforms in Ppn-inoculated tobacco leaves. Transcription levels of NtACS gene family members NtACS1 (left panel), NtACS2 (middle panel), and NtACS4 (right panel) at the indicated times after treatment with 1 μM PHS in Ppn-infected tobacco leaves. Transcription levels are expressed relative to the reference gene β-actin after qRT-PCR. Ethylene levels and relative mRNA expression levels are expressed as means ± SD. An asterisk indicates a significant difference in PHS-treated cases or PHS and Ppn inoculation co-treated cases from mock-treated cases (one asterisk (P < 0.05) or two asterisks at the same time point (P < 0.01)).

## Slide 6
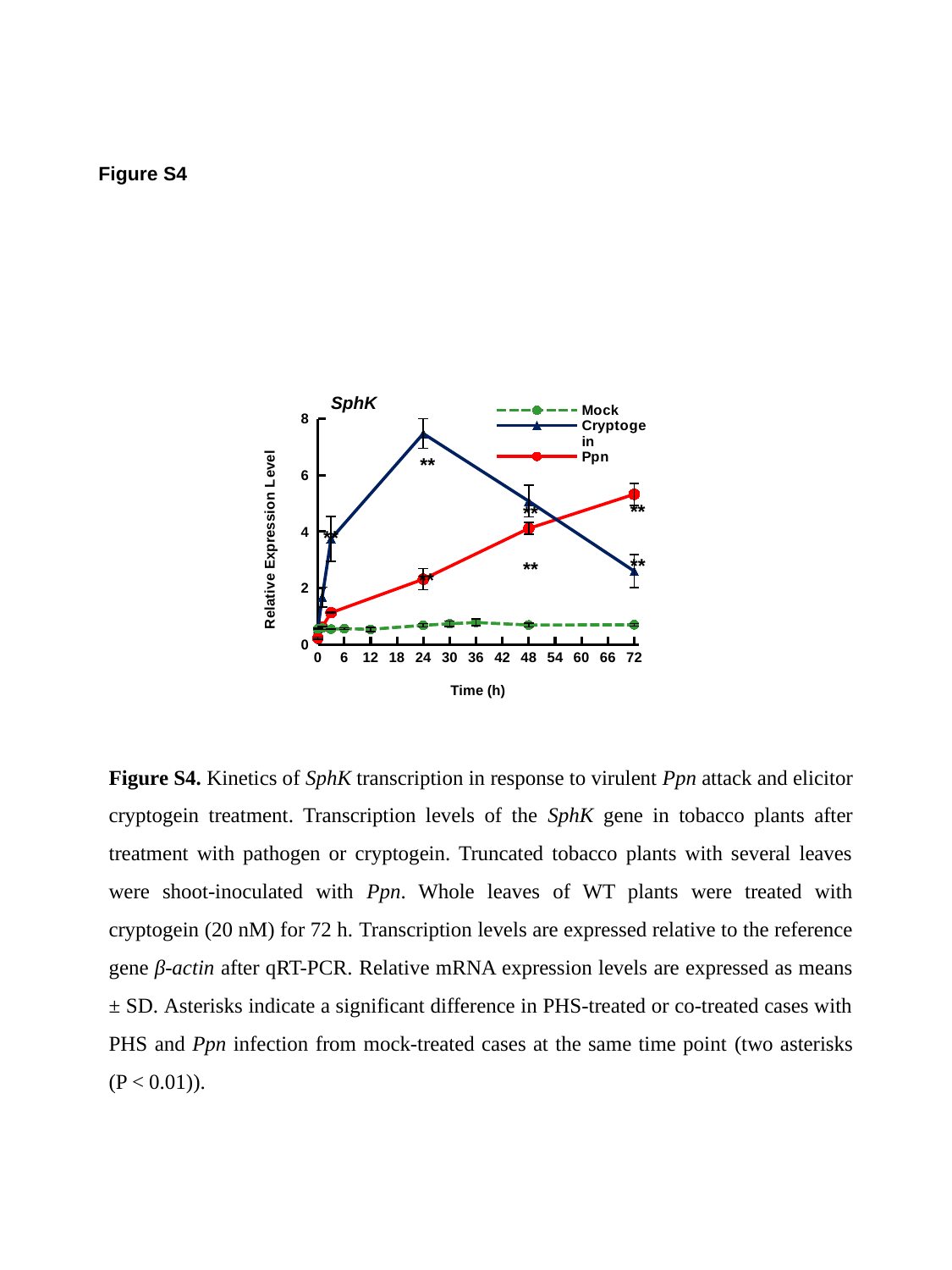

Figure S4
SphK
### Chart
| Category | Mock | Cryptogein | |
|---|---|---|---|**
**
**
**
**
**
Time (h)
**
Figure S4. Kinetics of SphK transcription in response to virulent Ppn attack and elicitor cryptogein treatment. Transcription levels of the SphK gene in tobacco plants after treatment with pathogen or cryptogein. Truncated tobacco plants with several leaves were shoot-inoculated with Ppn. Whole leaves of WT plants were treated with cryptogein (20 nM) for 72 h. Transcription levels are expressed relative to the reference gene β-actin after qRT-PCR. Relative mRNA expression levels are expressed as means ± SD. Asterisks indicate a significant difference in PHS-treated or co-treated cases with PHS and Ppn infection from mock-treated cases at the same time point (two asterisks (P < 0.01)).

## Slide 7
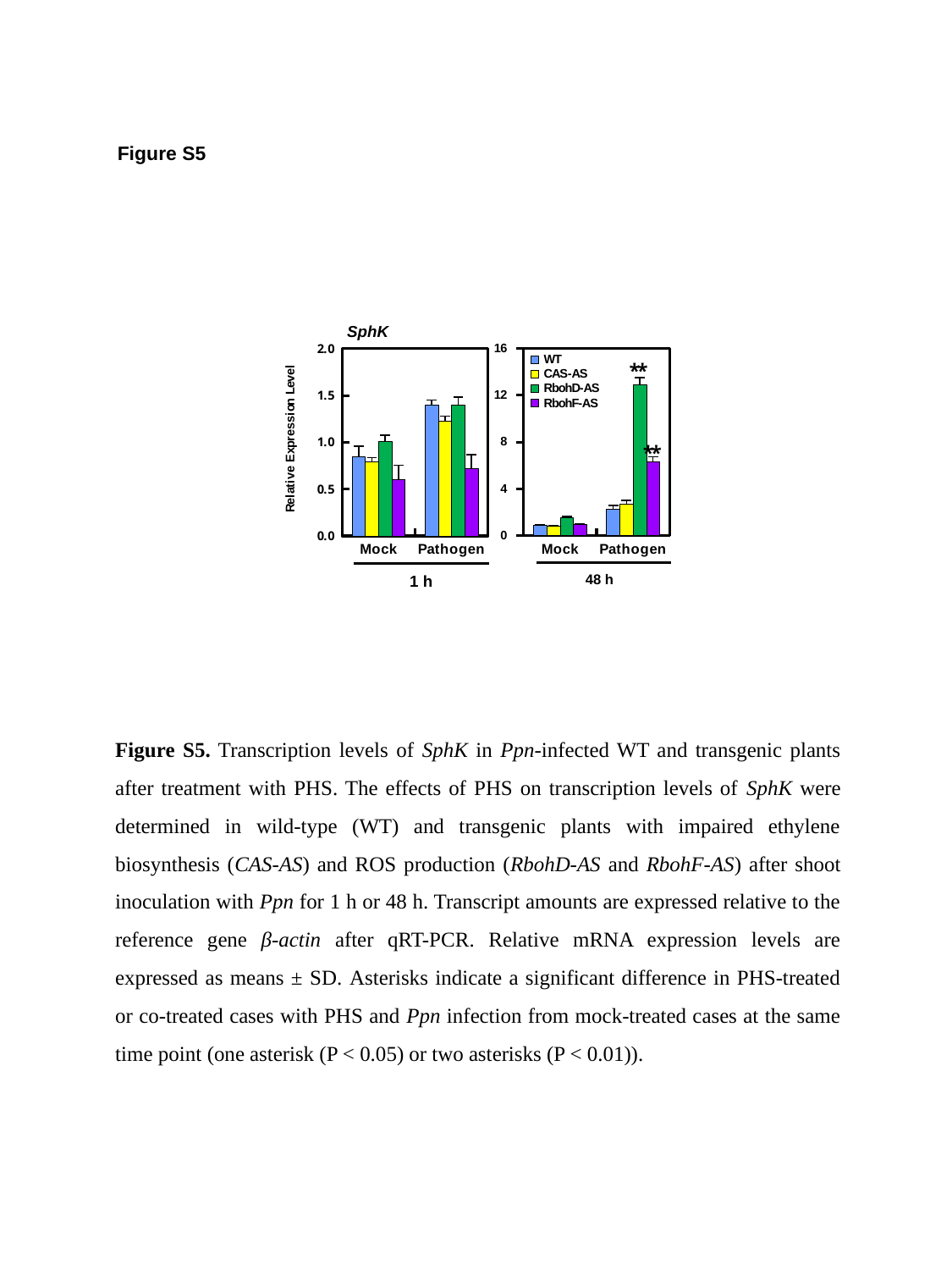

Figure S5
SphK
### Chart
| Category | WT | CAS-AS | RbohD-AS | RbohF-AS |
|---|---|---|---|---|
| Mock | 0.8676178298604565 | 0.7996139022559706 | 1.5213051853645359 | 0.9416120412089982 |
| Pathogen | 2.2732902538421693 | 2.6789657588805675 | 12.929618030531225 | 6.2984936642576494 |
### Chart
| Category | WT | CAS-AS | RbohD-AS | RbohF-AS |
|---|---|---|---|---|
| Mock | 0.8441485152821435 | 0.7894335506413435 | 1.0089515886294507 | 0.6021226582589252 |
| Pathogen | 1.4007541532640588 | 1.2227628479381094 | 1.4015889580708465 | 0.7185450395080213 |**
**
48 h
1 h
Figure S5. Transcription levels of SphK in Ppn-infected WT and transgenic plants after treatment with PHS. The effects of PHS on transcription levels of SphK were determined in wild-type (WT) and transgenic plants with impaired ethylene biosynthesis (CAS-AS) and ROS production (RbohD-AS and RbohF-AS) after shoot inoculation with Ppn for 1 h or 48 h. Transcript amounts are expressed relative to the reference gene β-actin after qRT-PCR. Relative mRNA expression levels are expressed as means ± SD. Asterisks indicate a significant difference in PHS-treated or co-treated cases with PHS and Ppn infection from mock-treated cases at the same time point (one asterisk (P < 0.05) or two asterisks (P < 0.01)).
